# I Can See It in Your Face. Affective Valuation of Exercise in More or Less Physically Active Individuals

**DOI:** 10.3389/fpsyg.2019.02901

**Published:** 2019-12-20

**Authors:** Ralf Brand, Lukas Ulrich

**Affiliations:** ^1^Sport and Exercise Psychology, University of Potsdam, Potsdam, Germany; ^2^Institute of Psychology, Humboldt University of Berlin, Berlin, Germany

**Keywords:** motivation, exercise, emotion, automatic facial expression analysis, Stroop effect

## Abstract

The purpose of this study was to illustrate that people’s affective valuation of exercise can be identified in their faces. The study was conducted with a software for automatic facial expression analysis and it involved testing the hypothesis that positive or negative affective valuation occurs spontaneously when people are reminded of exercise. We created a task similar to an emotional Stroop task, in which participants responded to exercise-related and control stimuli with a positive or negative facial expression (smile or frown) depending on whether the photo was presented upright or tilted. We further asked participants how much time they would normally spend for physical exercise, because we assumed that the affective valuation of those who exercise more would be more positive. Based on the data of 86 participants, regression analysis revealed that those who reported less exercise and a more negative reflective evaluation of exercise initiated negative facial expressions on exercise-related stimuli significantly faster than those who reported exercising more often. No significant effect was observed for smile responses. We suspect that responding with a smile to exercise-related stimuli was the congruent response for the majority of our participants, so that for them no Stroop interference occurred in the exercise-related condition. This study suggests that immediate negative affective reactions to exercise-related stimuli result from a postconscious automatic process and can be detected in the study participants’ faces. It furthermore illustrates how methodological paradigms from social–cognition research (here: the emotional Stroop paradigm) can be adapted to collect and analyze biometric data for the investigation of exercisers’ and non-exercisers’ automatic valuations of exercise.

## Introduction

Without a doubt, exercise is one of the behaviors that significantly contributes to better health and can prevent the development of diseases (e.g., Piercy et al., 2018). Exercise psychology has helped explaining why some people succeed more than others in changing their behavior to promote their own health. However, within exercise psychology, it is only recently that greater attention has been paid to the role of affective processes in health behavior change, in the maintenance of health behavior, and as a reason why people maintain unhealthy lifestyles (Ekkekakis et al., 2018; Ekkekakis and Brand, 2019).

Qualitative explorations (e.g., Ladwig et al., 2018), quantitative studies (e.g., Vanden Auweele et al., 1997), and review articles (e.g., Rhodes et al., 2009; Rhodes and Kates, 2015) have shown that negative affective judgment of exercise is an important reason that many people avoid exercise. Results from a representative survey of the resident population in Switzerland corroborate this finding (Lamprecht et al., 2014). Experimental studies provided evidence that heavy exercise intensity is often – and severe exercise intensity is inevitably – associated with negative affect (e.g., Ekkekakis et al., 2011). Another new branch of investigation has begun to highlight the role of positive and negative automatic evaluations of exercise in exercise motivation (e.g., Rebar et al., 2016; Schinkoeth and Antoniewicz, 2017; Chevance et al., 2019).

This very brief overview illustrates that the research interest in studies on affective aspects of exercise motivation (i.e., missing exercise motivation) has strongly increased in the past few years. Self-report methods have dominated the measurement of exercise-related reflective affective judgments (e.g., with the Feeling Scale, Hardy and Rejeski, 1989), and a few standard reaction time-based indirect tasks (mainly variants of the Implicit Association Test/IAT; Greenwald et al., 1998) were used for measuring automatic evaluations and associative processes. Although not all these reaction time-based measures reflect automatic processes in their purest form, they have become a method of choice in current exercise psychology research (Chevance et al., 2019).

In our view, continuous effort in testing the applicability of potentially powerful new measurement technology is justified. Therefore, it was one of this study’s aims to investigate the possibility of using state-of-the-art facial expression analysis technology to measure exercisers’ and non-exercisers’ automatic affective valuations of exercise. Addressing our second aim, whether spontaneous affective valuation occurs already when people are reminded of exercise, we focus a new theory, which will be briefly summarized first.

### Automatic Valuation of Exercise

Automatic valuation of exercise is the core construct in the Affective-Reflective Theory of physical inactivity and exercise (ART; Brand and Ekkekakis, 2018). This theory explains why, in the presence of an exercise-related stimulus, some people decide to change their current state of physical inactivity and become active, while others do not. The ART is a dual-process theory which assumes that an automatic (type-1) process provides the basis for a reflective (type-2) process which can follow if self-control resources are available.

One characteristic of the ART is that the type-1 process is considered to be essentially affective. The assumption is that, for example, the mere thought of exercise will activate automatic associations with exercise in memory and release an automatic affective valuation (i.e., a certain “gut feeling” with exercise; see also Damasio, 1996), which, if negative, can restrain a physically inactive person in his or her current state of physical inactivity.

Automatic affective valuations are theorized to form as a result of repeated experiences with exercise. Because exercise is not only a social but also a somatic stimulus, the automatic affective valuation includes core affective feelings of displeasure that arise directly from the body (e.g., the experience of shortness of breath during a run). More precisely, in the ART, affective valuation of exercise is conceptualized to arise from the somato-affective bonds that formed through one’s past experiences with exercise. These bonds are then re-actualized during the automatic processing of an exercise-related stimulus.

### Link Between Affect and Facial Expressions

Affect can be most broadly defined as a momentary pleasant or unpleasant state, that can be consciously accessible at any time, and has a non-reflective feeling at its core (Ekkekakis, 2013). It is a dimensional construct, which means that people can have more or less pleasant or unpleasant feelings, but never pleasant and unpleasant feelings at the same time. Affect is part of what people experience during moods and emotional episodes (Russell and Feldman Barrett, 1999). It can be described by its two dimensions affective valence and arousal (Russell, 1978).

There is evidence for a strong link between affective states and facial expressions. Congruency of self-reported affective states and spontaneous facial expressions (e.g., during affect-provoking short films or picture presentations) has often been documented (e.g., Bonanno and Keltner, 2004). Induced positive affect correlates with expressions of smiling and activity of the facial muscle zygomaticus major (a muscle which pulls the corners of the mouth back and up into a smile; e.g., Winkielman and Cacioppo, 2001), whereas increases in self-reported negative affect potentiate activity of the corrugator supercilii (a muscle which draws the brow down and together into a frown; e.g., Larsen et al., 2003).

Several studies showed that spontaneous (automatic) rapid facial muscle activity occurs approximately 500 ms after the display of pictures showing facial expressions (e.g., Dimberg et al., 2000, 2002). Further studies (using automatic facial expression analysis; see below) showed that spontaneous and deliberate smiles were comparable with regard to onset times, but that the amplitude of the onset phase (greater for deliberate smiles) and the ratio of duration to amplitude (higher for spontaneous smiles) differs (e.g., Cohn and Schmidt, 2004).

Recent neuro-imaging studies in non-human primates and humans provided evidence for emotion-to-motor transformation in neuronal pathways of emotional facial expressions (Gothard, 2014). Deliberate and spontaneous facial expressions are mediated by separate neural pathways however (e.g., Morecraft et al., 2007; Müri, 2016). There is evidence that initial facial expression is controlled by affect motor programs that can be triggered independently of conscious cognitive processes, i.e., without awareness of positive and negative eliciting facial expressions as stimuli (Dimberg et al., 2000).

### Measuring Affective Facial Expression

In the past, two approaches to the measurement of affective signs in facial expressions have proven especially successful in psychology. First, there is a long tradition in psychophysiology of differentiating pleasant and unpleasant states with facial EMG measures of activity over zygomaticus major and corrugator supercilii (Larsen et al., 2003). Second, there are classification systems to determine and taxonomize complex emotion-relevant facial movements and expression, of which the Facial Action Coding System (FACS; Ekman and Friesen, 1976) is the most popular one today.

FACS is an observer-based classification system, according to which trained coders view video-recorded facial movements in slow motion to code facial movements into action units (AUs). AUs refer anatomically to facial muscle activity (e.g., AU 12 as lip corner puller moved by the zygomatic major muscle) and/or head and eye movement (e.g., AU M68, upward rolling of eyes). The aim of the initial FACS was to decode basic emotions through a combination of simultaneously appearing AUs; an updated version allows for additional ratings on intensity or strength of expressions (Ekman et al., 2002). The system is widely used in different fields of research, with good to very good interobserver reliability (e.g., Sayette et al., 2001).

Very recently, computer systems with automatic coding software have been developed to make the decoding process significantly faster (i.e., perform real-time analysis) and even more reliably (Kulke et al., 2018). One of these methods, the Affdex algorithm (McDuff et al., 2010) as implemented in the iMotions^TM^ platform for biometric research was used in this study. Affdex relies on a normative data set based on manual initial codings of human FACS coders, and subsequent machine learning data enrichment with more than 6 million analyzed faces (Affectiva, 2018). The algorithm detects faces appearing in digital videos, and locates and tracks the movements of 34 facial landmarks on *x* and *y* coordinates. Vertical and horizontal differences between anchor (e.g., tip of the nose) and deformable (e.g., left and right lip corners) landmarks are algorithmically analyzed in a frame-by-frame analysis and mapped on a timeline. Each actual appearance of the face and the configuration of the facial features (e.g., lip corner puller moved 10 screen pixels upward accompanied by lifting of the cheeks) are statistically compared with a normative database to automatically classify facial expression.

### Stroop Interference

The attempt to evaluate participants’ affective valuation of exercise by observing their facial expressions will probably fail if one only waits for natural facial movements. People may not always make faces when they think of something; and probably not at all when they are participating in a psychological experiment and they know their face will be recorded. This study therefore used requested facial expressions which had to be shown as responses to stimuli in a Stroop test.

Stroop interference is a well-known and empirically robust phenomenon in cognitive psychology (MacLeod, 1991). In its basic form, a Stroop test asks participants to name the color in which a word is printed and ignore the meaning of the word thereby. Across trials, print color and word meaning can be compatible (e.g., say “green,” when the word *green* is printed in green) or incompatible (e.g., say “green” when the word *red* is printed in green). Stroop interference, causing slower and error-prone responses, occurs in incompatible trials, when print color and the word meaning do not match.

The mechanism behind the interference is that participants fall back on the default mental set (Besner et al., 1997) of processing the word to its semantic level despite instructions to the contrary. Because participants, at least in the described standard setup of the task, cannot deliberately suppress the slowdown and error tendency in incompatible trials, researchers suggested that there is a substantial automatic processing component (MacLeod, 1991). There is evidence, however, that at least some aspects of word recognition are not automatic (Besner et al., 1997).

The emotional Stroop test is a continuation of the original Stroop approach. Here, the participants are slower to name the font color of words when these words have affective meaning for them. It was shown, for example, that participants who can identify little with physical exercise (non-exerciser schematics) showed delayed response latencies for sedentary-lifestyle-related words but not for exercise-related words (Berry, 2006), and that college student binge drinkers responded more slowly to alcohol words than to neutral words (Hallgren and McCrady, 2013). Some studies used photographs with colored frames instead of colored word stimuli and have found comparable effects (e.g., with cocaine users; Hester et al., 2006). The mechanism behind emotional Stroop interference is that the affective stimulus creates an automatic attentional bias, which interferes with the response to the actual tasks (Cox et al., 2006; Sutton et al., 2007). While there is relatively consistent evidence for a deceleration of response times on negative stimuli (e.g., Pratto and John, 1991), results for an interference effect on positive stimuli is less consistent (e.g., Ruiz-Caballero and Bernandez, 1997). More recent research suggested that affective arousal contributes to the effect (Schimmack and Derryberry, 2005), and that state anxiety exacerbates the interference by further biasing attention toward the affectively salient stimuli (Dresler et al., 2009).

Taken in sum and related to the study here: Assuming that exercise-related stimuli trigger inter-individually different automatic valuations of exercise, we suggest that the detection of (partly automatic; Frings et al., 2009) attentional bias in a facial expression task can provide evidence for successful measurement of the underlying affective reaction.

### This Study

Against the background of the presented theory and evidence, we derived the following considerations for the study. We assumed that affective valuations of exercise differ according to the exercise volumes reported by the study participants (more positive affective valuation of exercise in those who exercise more), and that the respective affective valuation will automatically arise with the presentation of exercise-related pictorial stimuli.

We created a task similar to standard emotional Stroop tasks. Participants were asked to respond with a positive (smile) or negative (frown) facial expression depending on how picture stimuli were presented (upright or tilted, i.e., independent of what is shown in the photo). Onset times of these responses were measured with a software for automated facial expression analysis (incorporating the Affdex algorithm). Test trials therefore required participants to present a compatible or incompatible facial response to exercise-related stimuli. Our hypothesis was that participants will be faster in giving compatible responses (e.g., smiles after exercise-related pictures for those who exercise more, and frowns after exercise-related pictures for those who exercise less or do not exercise at all) than in giving incompatible responses (e.g., frowns after exercise-related pictures for those who exercise more, and smiles after exercise-related pictures for those who exercise less or do not exercise at all).

The major aim of this study was to demonstrate that attentional bias, hypothetically due to the participants’ inter-individually different automatic affective valuations of exercise, can be detected by means of automated facial action analysis. Using this technology (and combining it with the emotional Stroop paradigm to cover automatic aspects of the affective reaction) seemed promising because this would allow in the future the measurement of affective valuation to separate from the measurement of automatic associations, which can be addressed much more directly with an IAT.

To the best of our knowledge, this is the first study in which technology for automatic facial expression analysis is used within the framework of an emotional Stroop paradigm, and the first one to explore the potential of this technology for investigating affective-motivational processes related to exercise (or other health behaviors).

## Materials and Methods

In the following, test instruments and the exact procedure are presented first. Then the study sample is described. This seems reasonable because it allows to better understand and evaluate both aspects of the study (i.e., the test of the research hypothesis and the exploration of the applicability of automated facial action analysis as a method).

### Facial Action Analysis

#### Stimulus Material

Participants saw pictures that had been selected either as reminders of exercise (15 test stimuli) or as reminders of sedentary study work (15 control stimuli). Reminders of exercise depicted sports equipment (e.g., badminton racket with a shuttle, cardio machines in a fitness studio, and weight handles on a rack) and prototypical exercise situations (e.g., a swimmer who dives into the pool, a runner’s feet on a dirt road, and a person doing stretching exercises). Control pictures illustrated places for sedentary study work in the university (e.g., pictures from a library, a lecture hall, and a desktop with a computer and worksheets) and young people sitting and studying (e.g., writing hands, students talking at a table, and students sitting outside and reading). The photos did not show faces to avoid any obvious display of emotion in the picture and viewer mimicry effects.^1^

Some of the earlier studies on emotional Stroop interference conducted pre-tests for the used word stimuli in order to illustrate that participants actually understood what the word meant (e.g., whether words like *fit*, *muscle*, and *strong* are perceived in their meaning as relating to exercise, whereas words like *suburban*, *dog*, and *acoustic* are not; Berry, 2006). In studies with picture stimuli such pre-testing was often omitted (e.g., Hester et al., 2006), presumably because situations and contexts can be represented more unambiguously with pictures than with single words. We considered what was depicted with our pictures as obvious, and therefore refrained from pre-testing. However, in order to avoid any misunderstanding on the side of the participants, we presented all pictures prior to the experiment, so that participants were explicitly informed about the belonging of pictures to categories (i.e., exercise or sedentary work in the university), and we used response primes (words) explicitly referring to the category of each picture that would follow.

#### Picture Presentation and Experimental Task

All stimuli were 900 × 600 pixels in size, had a thin white frame (two pixels in size), and were presented centered against a black screen. Each picture was presented twice, once parallel to the edges of the monitor and once rotated to the right by one degree. The participants’ task was to produce either a smile or a frown as quickly as possible after stimulus presentation, depending on whether a picture was presented upright or tilted. The requested reaction was learned during a preceding practice block with 10 upright and 10 tilted gray rectangles (which were presented just like the pictures). Half of the participants, alternating in order of their appearance in the laboratory, learned to smile after upright and to frown after tilted pictures, whereas the other half learned to present displeasure after upright, and a smile after tilted pictures.

During the practice block (20 trials) the tilted and upright gray rectangles were presented in randomized order (within and between subjects), for 4 s each, with blank screens for 3 s between them. During the experimental block (60 trials) a 2-s response prime with either “physical exercise” or “study work” (white letters in the middle of the screen) preceded each stimulus to prefigure the rubric of the next picture, followed by a blank screen for 1 s, then the stimulus presentation for 4 s, and finally the blank screen for 3 s. Again, sequences of upright and tilted test and control pictures were fully randomized.

Participants were instructed to present the requested facial expression briefly and clearly, as quickly as possible after stimulus onset (i.e., as soon as a picture appeared on the screen), and relax their faces afterward until the next stimulus presentation. The participants received no feedback as to whether a facial response given was correct (smile vs. displeasure, depending on upright vs. tilted stimulus presentation), clear enough, or long enough.

#### Facial Action Decoding

Stimulus presentation and synchronized analysis of facial expressions were performed with the iMotions^TM^ platform for biometric research, which uses the Affectiva Affdex algorithm (McDuff et al., 2010). A high performance laptop computer with an external 22″ monitor was used for data collection. Participants’ faces were recorded continuously throughout the experimental task with an external HD webcam mounted on top of the external monitor.

The software recorded and continuously analyzed the configuration of the 34 facial landmarks during the presentation of stimuli, with a resolution of 30 frames per second (fps). The Affdex algorithm returns as a sequential score at frame rate in real time (and stores for later analysis) probabilistic results (0–100%) that indicate the likelihood of occurrence of defined facial actions (Affectiva, 2018). Importantly, the Affdex algorithm involves elements of artificial intelligence (non-parametric machine learning), so that more detailed information about the transformation of facial action data into scores for facial expression is not available. This is a major difference to the use of the FACS, which is based on parametrically defined coding rules. Recent research has shown that Affdex scores correlate highly with facial EMG measures, and that the algorithm is even better than this method in recognizing affectively neutral facial expressions (Kulke et al., 2018).

In this study, we used the Affdex facial expression score for affective valence. Positive affective valence is derived from defined changes in two facial landmarks (cheek raise, lip corner puller; one integrated valence score from 0 to 100), and negative affective valence from changes in eight facial landmarks (inner brow raise, brow furrow, nose wrinkle, upper lip raise, lip corner depression, chin raise, lip press, lip suck; one integrated valence score from −100 to 0).

#### Calculation of Test Scores

The main study variable was the onset time (in fps) of requested positive or negative facial expression after stimulus presentation onset. For every participant, single-trial reactions were grouped and averaged according to the four stimulus conditions “exercise-related stimulus and smile” (15 reactions), “exercise-related stimulus and frown” (15 reactions), “control stimulus and smile” (15 reactions), and “control stimulus and frown” (15 reactions). Then the time point (frame) at which an averaged valence score first exceeded the value 10 (for positive valence) or −10 (for negative valence) in the correct direction (as requested by the specific task) was marked in each participant’s data set and used for statistical analysis. In absence of comparable studies with facial expression analysis software, this threshold was chosen arbitrarily with the idea in mind to detect the very early signs of emerging (i.e., the requested) facial action. The two other coding rules were that the baseline value at stimulus onset had to be close to zero (<|10|), and that prior to the correct response no valence score increase of >10 must have occurred in the wrong direction. As a result, there were four preliminary scores for each participant (i.e., average onset times at which correct facial responses were given in the four stimulus conditions), which were calculated from the single trials with none of the three criteria violated.

Two facial action scores were then calculated and used for the main statistical analyses. *Express positive affect* is the ratio of average onset time for signs of positive valence in a participant’s facial expression after control stimuli, to average onset time for signs of positive valence after exercise-related stimuli. The higher this score is the easier it was for the participant to present a smile after exercise-related stimuli (i.e., the faster he or she did so, and the more this person might like exercise). The second score, *express negative affect*, is the ratio of average onset time for signs of negative valence in one’s facial expression after control stimuli, to average onset time for signs of negative valence after exercise-related stimuli. The lower this score is, the easier it was for participants to present a face of displeasure upon exercise-related stimuli (i.e., the faster they did so, and the less this person might like exercise).

Basically, the two scores represent reaction-time latencies for showing positive and negative affective facial expressions, which can be compatible or incompatible with the affective responses triggered by exercise-related versus control stimuli.

### Other Study Variables

#### Exercise Behavior

Exercise behavior was assessed with an item from the German version of the International Physical Activity Questionnaire (Hagströmer et al., 2006). Participants were asked with one question how much time they would usually spend doing moderate or vigorous exercise. Participants reported how many times they exercised each week (*number of exercise sessions*) and the average duration of these sessions (*exercise session duration*), by typing their answers in two separate free-text fields presented on the computer screen.

The two variables were measured as criteria that can be regressed to the two facial action scores. We expected more negative affective reactions from those who exercise less, and more positive affective reactions from those who exercise more. The reason why we refrained from calculating a total physical activity volume score by multiplying sessions by average duration was our experience in previous studies that it was easier for participants to correctly remember the number of exercise sessions than the respective durations. Calculating and using the multiplicative score would lead to multiplied measurement error, which can easily be avoided at this point.

#### Reflective Affective Evaluation of Exercise

This variable was measured (in German) with the single item “What is your feeling about exercise if you think about it now?” Participants gave their answer on a continuum marked with a −100 to +100 visual analog rating scale (at the left pole a frowning emoticon illustrated the descriptor “absolutely awful,” and at the right pole there was a smiling emoticon with “absolutely great”). By default, a slider lay on the neutral point 0 and could be moved with the computer mouse.

This variable, *reflective evaluation*, was taken as a side measure to support our hypothesis that the attentional bias evident in the experimental task and the participants’ reflective affective evaluation of exercise go in the same direction. We expected positive correlations with both exercise behavior and the facial action scores.

### Procedure

Test subjects were led into the laboratory and asked to sit down in front of a computer monitor. They were informed that they could abort the experiment at any time, without consequences. The investigator asked them whether they would permit the recording of their facial expressions during the experiment and anonymous storage of their data afterward. If the person agreed, the investigator explained the order of the tasks and what each would measure. Those willing to participate signed the prepared form to indicate informed consent.

All participants first worked on the experimental task, then gave the requested personal data (age, gender, subject of study at their university) and answered the questions on exercise behavior as well as their reflective affective evaluation of exercise.

After the completion of all tasks, the participants were debriefed about the goals of the study. It was again emphasized that all data would be stored anonymously, that the data would be used and analyzed exclusively for the purpose of the described investigation, and that it would not be accessible to third parties. They were asked to confirm their consent and, after they had agreed, they finally left the laboratory. This protocol was approved by the sport and exercise psychology ethics committee.

### Tests and Statistical Methods of Analysis

The study followed a cross-sectional design. Frequency counts, means, standard deviations, and Pearson correlation coefficients (one-sided tests, according to the study hypotheses) were calculated to describe the data set. For the same purpose, several *t*- and χ^2^-tests were applied. The main analysis then consisted of two linear regression models. One was calculated to predict “number of exercise sessions” from the two facial expression scores “express positive affect” and “express negative affect.” The other regression model was calculated for “session duration,” with the same two predictors. *A priori* calculation of the required sample size for detecting a medium effect size with linear multiple regression (fixed model, *R*^2^ deviation from zero; α error probability = 0.05, 1−β error probability = 0.80, three predictors) suggested a minimum of 77 cases for statistical analysis.

### Participants

Participants were recruited from students at a university campus. A total of 141 individuals were tested (*M*_age_ = 21.2 ± 3.3 years; 54 female), because informal pre-tests already indicated that the experimental task may be difficult to solve. Against the background of this larger sample, it is possible to estimate whether or how well the methodological paradigm presented may be adopted for further research.

The testing of our study hypothesis (differences in exercise behavior can be regressed to facial action scores) is based on the data of the 86 participants (*M*_age_ = 20.9 ± 2.4 years; 34 female) with complete datasets. This subsample was not significantly different from the whole group of tested individuals with regard to any of the investigated variables (Table 1).

**TABLE 1:** Sample sizes, means, and standard deviations for all main study variables and correlations.

	**Sample as tested**			**Sample as analyzed**						
**Variables**	***n***	***M***	***SD***	***n***	***M***	***SD***	**1**	**2**	**3**	**4**
1. Reflective evaluation	141	55.21	37.33	86	56.37	38.22				
2. Express positive affect	110	0.97	0.13	86	0.98	0.12	0.28^∗∗^ [0.07, 0.45]			
3. Express negative affect	108	0.99	0.18	86	0.99	0.16	0.09 [−0.12, 0.30]	−0.06 [−0.27, 0.15]		
4. Number of exercise sessions	141	3.61	2.39	86	3.80	2.31	0.58^∗∗^ [0.42, 0.71]	−0.10 [−0.31, 0.11]	0.26^∗^ [0.05, 0.45]	
5. Average session duration	141	68.90	38.43	86	67.97	33.83	0.47^∗∗^ [0.29, 0.62]	−0.13 [−0.33, 0.08]	0.23^∗^ [0.02, 0.42]	0.58^∗∗^ [0.42, 0.70]

*^∗^*p* < 0.05, one-tailed; ^∗∗^*p* < 0.01, one-tailed; 95% CI in square brackets.*

Sixty-four percent (*n* = 55) of the participants in the subsample were majoring in sport and exercise and the median number of exercise sessions per week in this group was 5 (median 50% quantile between 4 and 6), with a mean session duration of 76.8 min (SD = 20.3). From the remaining 36% participants, 21 were leisure-time exercisers (*Md*_sessions_ = 2; interquartile range from 1 to 4; *M*_duration_ = 77.1, SD = 33.7; nine female) and 10 were non-exercisers (i.e., reported zero weekly minutes of exercise per week; nine female).

Irrespective of the subject of study at their university, male participants reported significantly more exercise sessions per week [male: *M* = 4.38, SD = 1.98; female: *M* = 2.91, SD = 2.51; *t*(84) = 3.03, *p* = 0.003] and longer average session duration [male: *M* = 79.04, SD = 24.36; female: *M* = 49.85, SD = 37.12; *t*(84) = 4.41, *p* < 0.001]. There were significantly more males (*n* = 39) than females (*n* = 16) in the group of sport and exercise majors, χ^2^(1) = 7.0, *p* < 0.001.

## Results

### Task Difficulty

Working with the facial expression task was not easy for the participants. Coding the participants’ (*N* = 141) single-trial reactions according to the three criteria for identifying correct and unambiguous test responses (see first paragraph in the “Calculation of Test Scores”) showed a range of 0–8 (out of 15 per stimulus condition) false responses (e.g., a frown shown instead of the requested smile). We decided that >20% false or missing reactions per condition may seriously affect test value reliability, and that no aggregated score would be calculated then.

This rationale allowed to calculate at least one facial action score (out of four possible scores) for four out of five participants (between 81 and 84% of participants). From this, the composite scores “express positive affect” and “express negative affect” could be derived for 78 and 77% of all participants. Both scores were available for 61%, that is the 86 participants of the study sample. The statistical exploration of possible correlations between errors in task completion and the other variables (e.g., age, gender, exercise behavior, and reflective evaluation; Table 1) did not lead to significant results.

### Facial Action

On average, the first indications of a requested facial response appeared 27.01 frames (SD = 4.5), or 903 ms, after stimulus onset. This is almost half a second later than measured in other studies with automatic facial expression analysis tools (in which the response, however, was not measured in an emotional Stroop test; Cohn and Schmidt, 2004). Mean reaction times for initiating positive and negative facial expressions were correlated with medium effect size, *r*(86) = 0.29, *p* = 0.007. In total, negative facial expressions (*M* = 25.98, SD = 5.34) were produced slightly faster (i.e., 69 ms on average) than positive facial expressions (*M* = 28.05, SD = 5.96), *t*(85) = 2.84, *p* = 0.006, *d* = 0.37. Further descriptive statistics for and bivariate correlations between all main study variables are given in Table 1.

The *F*-test for the regression model with *number of exercise sessions* as the criterion and the three predictors “express positive affect,” “express negative affect,” and “reflective evaluation” was significant, *F*(3,82) = 16.72, *p* < 0.001, *R*${}_{\mathrm{adj}.}^{2}$ = 0.36. While “express positive affect” was not significant, “reflective evaluation” (β = 0.57, *p* < 0.001) and “express negative affect” (β = 0.21, *p* = 0.022) were significant predictors in this model. Participants who were faster with initiating negative facial expressions on exercise-related stimuli relative to initiating negative facial expressions on control pictures reported fewer exercise sessions per week (and those who were slower reported to exercise more often). With regard to effect size, the regression model shows that an increase of the test value in the predictor “express negative affect” by 0.25 units – which means that the difference between the average onset time for signs of negative valence after exercise-related stimuli and onset time for signs of negative valence in one’s facial expression after control stimuli is approximately halved – results in approximately 1 more exercise session per week (*B* = 2.89).

The *F*-test for the regression model with *average session duration* as the criterion and the three predictors “express positive affect,” “express negative affect,” and “reflective evaluation” was significant as well, *F*(3,82) = 9.38, *p* < 0.001, *R*${}_{\mathrm{adj}.}^{2}$ = 0.23. Again, “express positive affect” was not significant, and only “reflective evaluation” (β = 0.44, *p* < 0.001) and “express negative affect” (β = 0.19, *p* = 0.046) significantly predicted “average session duration.” Participants who were faster with initiating negative facial expressions on exercise-related stimuli relative to initiating negative facial expressions on control pictures reported lower average duration of exercise sessions (and those who were slower reported longer exercise sessions). In terms of effect size, this indicates that an increase in the difference between the average onset time for signs of negative valence after exercise-related stimuli and onset time for signs of negative valence in one’s facial expression after control stimuli by about 75% is equivalent to about 20 min shorter exercise session duration (*B* = 39.32). This effect is probably less remarkable than the one observed for “number of exercise sessions.”

More detailed results for both regression models are given in Table 2. In both models, independence of the predictors was demonstrated with collinearity statistics (all tolerances < 0.90, all VIFs < 1.12). Durbin–Watson test values of 1.85 and 1.95 indicated independence of the residuals. Approximate normal distribution of the residuals was checked graphically and found sufficient by histograms. The same was true for linearity of relationships between the included variables, as checked with partial regression diagrams. The homoscedasticity of residuals was inspected and affirmed with scatterplots of the *z*-transformed predicted values and *z*-transformed residuals. Supplementary regression analyses showed that the statistical relevance of “express negative affect” as predictor of the number of exercise sessions and average session duration was not confounded by the age and gender of the participants.

**TABLE 2:** Results from two separate multiple linear regression models.

**Criterion (regressors)**	***B* (95% CI)**	**SE *B***	**β**	***t***	***p***
**Number of exercise sessions**					
(Reflective evaluation)	0.04(0.02,0.05)	0.01	0.57	6.29	0.001
(Express positive affect)	1.04(−2.36,4.43)	1.71	0.05	0.61	0.546
(Express negative affect)	2.89(0.43,5.35)	1.24	0.21	2.33	0.022
**Average session duration**					
(Reflective evaluation)	0.38(0.21,0.56)	0.09	0.42	4.19	0.001
(Express positive affect)	−5.96(−57.94,48.83)	27.74	−0.02	−0.22	0.867
(Express negative affect)	39.32(0.64,77.99)	19.44	0.19	2.02	0.046

## Discussion

The overall aim of this study was to demonstrate that participants will show inter-individually different affective valuations of exercise (Brand and Ekkekakis, 2018) if they are shown visual stimuli that remind them of exercise. We took advantage of a setup, in which the hypothesized affective response (more positive in those who exercise more, and more negative in those who exercise less) would interfere with performance in an unrelated task (react to the tilted versus upright presentation of the stimulus) because of attentional bias. Emotional Stroop tasks like this have been used to point out features of automaticity in the preceding affective reaction (Pratto and John, 1991). By asking the subjects to respond with facial actions (smile or frown) that were more compatible or incompatible with their hypothesized affective valuation, we have chosen an unusual response modality for our task however. With the help of facial expression analysis software this study showed that those who exercise less were able to react faster with a frown to exercise-related pictures than those who exercise more often. The finding that those who exercise less also reported more negative reflective evaluations indicates that the faster facial response to exercise-related stimuli is likely to be based on the compatible affective valuation. The inverse effect that those who exercise more would be able to smile faster after exercise-related pictures could not be shown however.

### Automaticity of the Affective Response

The extent to which an experiential process or behavior is unintentional, occurs outside of awareness, is uncontrollable, and is efficient in its use of attentional resources, defines the degree to which the process or behavior is assumed to be more or less automatic (Bargh, 1994). Previous studies on the degree of automaticity in Stroop interference usually referred to word color Stroop tasks, and indicated that activation of the semantic level (i.e., understanding the meaning of a word) during reading should be considered partly automatic (because of the possibility to experimentally curtail the interference effect; e.g., Besner et al., 1997). Similar conclusions have been drawn for standard (color naming interference in word tasks) emotional Stroop tasks (Phaf and Kan, 2007; Frings et al., 2009). Therefore, the degree to which the effect observed here, although measured with a read-free task and with participant reactions in the form of facial expressions, was automatic is debatable as well.

Our argumentation on this differs to some extent from that in other studies. We suggest that the effect shown is based on a *postconscious automatic process* (Bargh, 1994) that relied on the residual activation of the consciously processed response prime (participants read whether the next picture belonged to the rubric “physical exercise” or the control category “study work”), in combination with the intentionally irrepressible reaction to the personally more or less relevant (we suspect liked or disliked) concept of exercise. This interpretation corresponds well with previous findings according to which participants were unable to suppress corrugator (frown) and zygomaticus (smile) facial muscle response patterns (EMG measurement) on affective stimuli, despite the instruction to avoid any facial movements (Dimberg et al., 2002).

Nevertheless it is possible that, for example, delays in detection or perception of stimuli, general action speed, or general tendencies to more often initiate positive or negative facial expressions had an effect on the observed reaction times of the participants.

Another question is whether the interference shown is really due to an *affective* reaction. Although emotional Stroop tasks assume exactly this, the present study cannot give a definite answer to this question. But, there are arguments to support this view. One is based in the modality of the measured reaction (which was how fast participants were able to initiate affect-related facial action), and it is rooted in neurobiology where it was possible to find evidence for emotion-to-motor transformation in bidirectional, relatively unmediated, amygdalo-motor pathways of emotional facial expressions (e.g., Gothard, 2014).

An additional argument has already been touched on above. Previous studies have shown that decelerated initiation of conscious facial action (smiling vs. frowning) can be experimentally induced when an incongruent facial response to an affective facial expression is requested (e.g., frowning at happy faces and smiling at angry faces; Dimberg et al., 2002). Against the background of the numerous empirical findings that the affective judgments of exercise differ between individuals (Rhodes et al., 2009), the step is short to the assumption that the exercise pictures we used elicited inter-individually different affective reactions as well (Schinkoeth et al., 2019).

Finally, our findings with the facial action score “express negative affect” correspond to the direction of the correlation measured between the participants’ reflective affective evaluation and self-reported exercise behavior. Those who did less exercise or none at all indicated more negative reflective affective evaluation of exercise (and those who exercised more indicated a more positive one), that is, in a measure independent of the facial expression task.

All in all, against the background of these considerations, we suggest that the affective reaction measured experimentally in our setup contains features of automaticity and is at least close to the construct of affective valuation, as it is defined in the ART (Brand and Ekkekakis, 2018).

### Interference With Negative Versus Positive Facial Expression

We have shown that only the score “express negative affect,” but not the score “express positive affect,” allowed the prediction of exercise behavior. One of the most compelling explanations for this result can be found in known properties of Stroop interference and, in our opinion, may partly result from a sampling error. In our study, people who exercised a lot and regularly were in the majority compared to those who avoided exercise. It is known from previous studies with emotional Stroop tasks that there is a deceleration of response times especially for negative stimuli (Algom et al., 2004; Pratto and John, 1991), but not as much for positive stimuli. Moreover, Stroop interference only appears in incongruent trials. Responding with a smile to exercise-related stimuli may thus have been the congruent response for the majority of our participants. The “express positive affect” score might have been less informative than the “express negative affect” score therefore.

An alternative explanation for a better predictive value of the negative response beyond this sampling effect (majority of exercisers) may be found in the neuronal pathways of affect. There is an important role of the amygdala and the brainstem in the activation of affective memories in the absence of higher cognitive processes (e.g., Costafreda et al., 2008), and there were significant (time) restrictions in our experimental task. It is known that amygdalo-subcortical areas contribute especially to negative affective responses (e.g., Janak and Tye, 2015). Together these factors may have caused faster and more intense negative affective responses in individuals who disliked exercising than positive affective responses in persons who liked exercising. This difference could also explain the superior informative value of the negative facial expression.

Finally, it cannot be ruled out that it might have been difficult for the participants to display positive affect on their faces, because they felt that the whole study situation was unpleasant. In view of the alternative explanations outlined above, we consider this to be less likely however.

### Study Limitations

Among the limitations of the study are, first, that participants were young in age and all recruited from a university campus. Subsequent studies should aim for more heterogeneous study samples. Second, we used self-reported exercise, namely how people think about their behavior, as the external criterion, and not a more objective measure of it. Future studies might employ, for example, multi-day accelerometry or behavioral tasks that can be observed in the laboratory for a more objective assessment of the participants’ exercise behavior. Furthermore and third, we do not know whether the picture material we used was the most appropriate for activating the hypothesized inter-individually different affective valuations on the side of the participants. Although we are not sure that there can be pictures that equally evoke the same mental model about exercise in all study participants, such stimulus properties could be pre-tested anyway. Fourth, our decision to use “study work” as a control category (with the expectation that this physically inactive behavior would be one that activates comparatively more negative affect, especially in sport and exercise majors) was arbitrary. Similar to IATs, it is possible that a single-target variant of our task could offer advantages over variants with comparison categories (Bluemke and Friese, 2008); although on the other hand there is evidence that single target variants may provide lower test–retest reliability (Chevance et al., 2017). Fifth, calculating ratio scores based on manifest variables results in the multiplicative combination of sources of measurement errors into single variables. This may have adversely affected test accuracy in our study. Finally, sixth, it could be argued that the attentional bias induced with emotional Stroop tests is dependent on inter-individually different inhibitory control resources (Cothran and Larsen, 2008). Although we are not aware of research conducted with (variants of) the emotional Stroop test in which this has already been done, further studies might want to control for this variable.

### Recommendations for Further Studies

In our study, no score was calculated for about 40% of the participants because they did not show the requested (but a wrong) facial reaction. Obviously, the task was too demanding for many participants. Should the experimental setup be further developed toward a test suitable for psychometric analysis at the individual level, it is certainly necessary to include more practice sessions for the participants, so that the task (smile or frown after upright/tilted pictures) is better mastered and the number of errors can decrease. Calculating test scores according to signal detection theory (e.g., Hautus, 2015) or the exploitation of mixed-effects model scores (e.g., Wolsiefer et al., 2017) may offer different approaches to data analysis, with superior possibilities.

Our study focused high intensity physical activities, i.e., exercise especially. This was done because we have learned from previous (own) studies that if people are to remember and report their usual level of physical activity, they remembered moderately (or higher) intensive exercise sessions better than less intensive ones. In addition, physical sensations (and resulting experience) during moderate and high intensive exercise sessions may leave deeper traces in (embodied) memory compared with those from less intensive episodes (Brand and Ekkekakis, 2018; Ekkekakis and Brand, 2019). With the focus on moderately and higher intensive forms of exercise we wanted to make it more likely to detect the expected valuation effect in the laboratory. Future studies should examine whether similar effects can also be demonstrated for lighter forms of physical activity. All the more so as our experimental setup referred to physically inactive behaviors as a control condition, it might be interesting to test whether automatic facial expression analysis can detect individuals’ (positive and negative) automatic affective valuations of sedentary behaviors as well (Cheval et al., 2018).

Finally, there are alternative algorithms that can be used for facial action analysis (e.g., Baltrušaitis et al., 2018). These should also be tested.

## Conclusion

We have adapted an established methodological paradigm from social–cognition research in this study (the emotional Stroop tasks) to collect behavioral data beyond the self-report of one’s own experience. The proposed paradigm is quite specific and may be suboptimal for other questions in the same research area. However, this study has taken advantage of one of the new opportunities offered by automatic facial expression software. This technology, if used wisely, offers completely new approaches to the investigation of new research questions. For example, the skillful use of the method might allow for studying participants’ affective responses during exercise, that is, without asking questions that probably disturb the physical activity and thereby affect their momentary affective state.

In addition, we believe that the results of our study contribute evidence for one of the central claims of the ART (Brand and Ekkekakis, 2018). In line with results from another recent study (e.g., Schinkoeth et al., 2019) our results suggest that individuals show inter-individually different affective valuations on exercise-stimuli, depending on whether they like and regularly engaged in exercise in the past. We therefore continue to assume that the negative affect that exercise abstainers often experience already when they start thinking about exercise provides a significant *restraining force*, making it difficult for them to become physically active. The same goes for the reverse: positive affect associated with the thought of exercise may be the *driving force* for those who exercise with ease. We hope that this theoretical idea, perhaps with a method similar to the one presented here, will lead to more empirical research in the future.

## Data Availability Statement

The datasets generated for this study are available on request to the corresponding author.

## Ethics Statement

Ethical review and approval was not required for the study on human participants in accordance with the local legislation and institutional requirements. The patients/participants provided their written informed consent to participate in this study.

## Author Contributions

All authors listed have made a substantial, direct and intellectual contribution to the work, and approved it for publication.

## Conflict of Interest

The authors declare that the research was conducted in the absence of any commercial or financial relationships that could be construed as a potential conflict of interest. The reviewer, MW, declared a shared affiliation, with no collaboration, with one of the authors, LU, to the handling Editor at the time of review.

## Footnotes

1 All photos used are available on request from the authors.
